# Modelling and optimisation of ultrasound-assisted extraction of roselle phenolic compounds using the surface response method

**DOI:** 10.1038/s41598-023-27434-5

**Published:** 2023-01-07

**Authors:** Abdoudramane Sanou, Kiessoun Konaté, Kaboré kabakdé, Roger Dakuyo, David Bazié, Sama Hemayoro, Mamoudou Hama Dicko

**Affiliations:** 1 Laboratory Biochemistry, Biotechnology, Food Technology and Nutrition (LABIOTAN), Department of Biochemistry and Microbiology, University Joseph KI-ZERBO, 03 B.P. 7021, Ouagadougou, Burkina Faso; 2 Applied Sciences and Technologies Training and Research Unit, University of Dedougou, B.P.176, Dedougou, Burkina Faso; 3 Laboratory of Biochemistry and Chemistry Applied (LABIOCA), University Joseph KI-ZERBO, 09 P.O. Box 848, Ouagadougou, Burkina Faso

**Keywords:** Biochemistry, Biological techniques, Biophysics

## Abstract

Extracts from *Hibiscus sabdariffa* L. (roselle) have been used traditionally as a food, in herbal medicine, in hot and cold beverages, as flavouring or coloring agent in the food industry. In vitro and in vivo studies and trials provide evidence, but roselle is poorly characterised phytochemically due to the extraction processes. The optimization of the extraction of phenolic compounds and their antioxidant activities is still a hot topic. In this study, the effect of solute/solvent ratio (33, 40 and 50 mg/mL), extraction temperature (40, 50 and 60 °C) and extraction time (30, 60 and 90 min) was evaluated through the content of phenolic compounds and antioxidant activity. A response surface methodology through a Box–Behnken design was applied and model fit, regression equations, analysis of variance and 3D response curve were developed. The results showed that TPC, TFC, DPPH and FRAP were significantly influenced by temperature, extraction time and solvent/solute ratio. Thus, TPC, TFC, DPPH and FRAP varied from 5.25 to 10.58 g GAE/100 g DW; 0.28 to 0.81 g QE/100 g DW; 0.24 to 0.70 mg/mL; 2.4 to 6.55 g AAE/100 g DW respectively. The optimal experimental condition (41.81 mg/mL; 52.35 °C and 57.77 min) showed a significant positive effect compared to conventional methods. The experimental values at this extraction condition show that this optimization model is technologically, financially and energetically viable as it requires a reasonable concentration, time and temperature.

## Introduction

*Hibiscus sabdariffa* (roselle) is a herbaceous plant that is very rich in bioactive compounds and has interesting therapeutic properties^1^. Indeed, several phytochemical screenings have shown that the therapeutic potency of roselle could be attributed to the qualitative and quantitative content of bioactive compounds^2^. These main components compounds^3^ include phenolic acids, flavonoids, anthocyanins, etc. Thus, the therapeutic properties attributed to roselle are antioxidant, anti-inflammatory, anti-carcinogenic, anti-obesity, anti-diabetic, antibacterial and anti-hypertensive effect^4, 5^. However, the extraction of these bioactive compounds is strongly influenced by the extraction parameters such as solvent/solute ratio, temperature and extraction time^6, 7^. Indeed, the conventional methods: decoction, maceration and infusion have limitations such as thermal degradation of some thermolabile biomolecules and long extraction time^8^. Nowadays, several methods including ultrasonic assisted extraction (UAE) are being developed to improve the quality and quantity of bioactive compounds in plants^9^. UAE method is a fast and reliable method for the extraction of primary and secondary metabolites^10^. In this method, the intensity of the ultrasound energy creates an additional vibration that damages the cell walls in the vegetative tissues of the sample, which facilitates the diffusion of the solute in the tissue to the solvent and a good extraction of the bioactive compounds^11^. In addition, ultrasound-assisted extraction can be used with polar and non-polar solvents at different temperatures^12^. Furthermore, it is an emerging potential ecological processing method due to the preservation of structural and molecular properties and low environmental pollution^13^. For any extraction technique, the optimization of the phenolic and antioxidant profile requires the adjustment of the extraction parameters. To this end, over the last decade, the use of surface response methodologies (RSM) has been applied in order to optimize the extraction conditions according to the desired objectives, thus improving the efficiency of advanced extraction technologies^7^. RSM is a sum of statistical and mathematical techniques, successfully used to develop, improve and optimize processes^14^. It is rapid and provides sufficient information about multiple independent variables and interactive effects of variables on responses^15^. In this study, we investigated the optimal extraction conditions for roselle through response surface methodology (RSM). RSM models, such as the Box-Behnken design (BBD), central composite design (CCD), and three-level full factorial design (TFFD), have been widely used to show interactions with one or more response variables and set the optimal extraction conditions.

## Materials and methods

### Plant material

Roselle calyxes were collected in Bobo-Dioulasso (Burkina Faso) in December 2020, from producers who kindly accepted sampling for research purposes. The calyxes used are not endangered species and the principles of experimental research and field studies on plants, including the collection of plant material, were in accordance with relevant institutional, national and international guidelines and legislation for plant material research. Subsequently, the collected samples were labelled, stored in a cooler and transported to the laboratory. After drying, the samples were finely ground using a RETSCH-GmbH grinder with an integrated 0.3 mm diameter sieve for the different extractions. In addition, an authentication was carried out by the services of Dr Mohamed CISSE, botanist at the Joseph Ki-Zerbo University, and a specimen with the reference 18015/6975/2020/SA was assigned in the herbarium of the Joseph Ki-Zerbo University.

### Conventional extraction approaches

Three conventional extraction methods (decoction, infusion and maceration) were carried out^16^. Indeed, the decoction consisted in boiling 10 g of finely crushed calyxes in 200 mL of distilled water during 1 h on a hot plate. The infusion consisted in pouring 200 mL of water previously boiled on 10 g of roselle powder during 5 min. Finally, a maceration at room temperature (10 g of roselle powder mixed in 200 mL of distilled water) was performed after 24 h. After these different extractions, the extracts were successively cooled (except for the maceration), filtered, lyophilized and stored at room temperature for the various analyses.

### UAE approach

In this study, ultrasonic extraction was performed according to the method used by Bimark et al.^16^ with some modifications. For this purpose, a 1.8 L capacity ultrasonic system (15 cm × 14 cm × 10 cm depth) (model 1510E-MT, Branson, USA) at a fixed frequency of 50 Hz and variable temperature was used. Thus, the extraction of bioactive compounds from roselle consisted of dissolving roselle powder in distilled water according to the variables of the UAE process, including temperature, solute-to-solvent ratio and extraction temperature, as shown in Table 1. In particular, the temperature was controlled using a water bath around the extraction flask. The extract obtained was then cooled, filtered, freeze-dried and stored at room temperature for the different analyses. Finally, the predicted optimal condition required an additional test to be performed in the optimized condition (41.81 mg mL^−1^; 52.35 °C and 57.77 min).

**Table 1:** Coded levels and actual values of the independent variables.

Independant variable	Levels		
	− 1	0	1
Temperature (°C)	40	50	60
Solid to solvent ratio (mg/mL)	33	40	50
Extraction time (min)	30	60	90

### Determination of total phenolic content (TPC)

Total phenolic content (TPC) of roselle extracts was determined using the Folin-Ciocalteu method as described by Singleton et al.^17^ with slight modifications. Briefly, 200 μL of each extract was added to 400 μL of diluted phenolic reagent (0.2 N) and vortexed for 1 min. Then 1 mL of Na_2_CO_3_ (75 g/L) was added to the mixture and incubated for 2 h at room temperature. Finally, the absorbances of the extracts were read at 765 nm compared to the gallic acid as a standard. The TPC was thus expressed as mg gallic acid equivalents per 100 g (mg GAE/100gDW).

### Determination of total flavonoids content (TFC)

The total flavonoid content of the extracts was determined according to the method described by Meza et al.^18^ with slight modifications. Briefly 500 µL of roselle extract and 500 µL ml of AlCl_3_ were thoroughly mixed and left at room temperature for 15 min and the absorbances were read at 415 nm using a spectrophotometer. The TFC content was estimated from the quercetin calibration curve and the result was presented as mg EQ/100mgDW.

### DPPH antioxidant assay

The approach consisted in determining the radical scavenging activity (RSA) of the extracts at different concentrations. Then, the IC_50_ corresponding to the concentration (mg mL^−1^) of the extract to trap 50% of the free radicals and thus translating their antioxidant activity. The capacity of the extracts to trap the DPPH (2,2-diphenyl-1-picrylhydrazyl) radical was thus evaluated as previously described^19^. The averages of three values were expressed as µg EAA/100 mgDW.

### Ferric reducing antioxidant power (FRAP)

The FRAP (Ferric Reducing Antioxidant Power) method is based on the reduction of ferric ion (Fe^3+^) to ferrous ion (Fe^2+^). The variant of Hinneburg et al.^20^ was used to evaluate the iron reducing power of roselle extracts. To a test tube containing 0.5 mL of sample solution (50 mg/mL), were added 1.25 mL of phosphate buffer (0.2 M, pH 6.6) and 1.25 mL of potassium hexacyanoferrate [K_3_ Fe (CN)_6_] 1% in water. The mixture was heated to 50 °C in a water bath for 30 min. Afterwards, 1.25 mL of trichloroacetic acid (0.1%) is was added and the mixture is centrifuged at 2000 rpm for 10 min. To 125 μL of the supernatant, 125 μL of distilled water and 25 μL of freshly prepared 0.1% FeCl_3_ in water are added to 96-well wells. A blank without sample is prepared under the same conditions. The reading is taken at 700 nm against an ascorbic acid standard curve (200 mg/L in distilled water). The iron reducing potential of the extracts is expressed as mg EAA/100gDW.

### Experimental design

In this study, a Box–Behnken design (BBD), three level full factor design (TFFD) and response surface methodology (RSM) were used to assess the relationship between three independent factors and the dependent variables and identify the optimal levels of the independent variables for the dependent variable (responses)^21,22^. Three independent variables, solute/water ratio (33 mg mL^−1^; 40 mg mL^−1^ et 50 mg mL^−1^), ultrasound extraction temperature (B) and time of extraction (C) were selected and each factor was associated with three distinct coded levels (Table 1). Total phenolic content (Y1), total flavonoid content (Y2), FRAP value (Y3) and DPPH scavenging activity (Y4) were chosen as dependent variables (Table 1). The experimental data were fitted to the following second order polynomial model and regression coefficients were obtained. The proposed generalized second-order polynomial model for the response surface analysis was given as follows:

$${\text{Y}} = \beta_{0} + \mathop \sum \limits_{i = 1}^{n} biXi + \mathop \sum \limits_{J = 1}^{n = 1} \mathop \sum \limits_{j = 2}^{n} bijXiXj + \mathop \sum \limits_{i = 1}^{n} b_{ii} X_{i}^{2}$$

where Y is the response variable, Xi and Xj are the independent variables and k is the number of tested variables (k = 3).

### Statistical analysis

Response surface methodology (RSM) is used to find the optimal mode of interaction between factors and to estimate the optimal conditions for the extraction process with the minimum number of experiments required. The RSM software determines the effects of independent variables on the processes, individually or in a set of factors. A Box–Behnken design consisting of 17 experiments with 5 replications of the central point was applied for the statistical analysis. All analyses were performed in triplicate (to calculate the reproducibility of the process) and the results were expressed as mean and standard deviation. For data analysis, Expert Design software version 7 was used for analysis of variance and response surfaces and regression equations and determination of optimum parameters. Finally, Graph-PadPrism 9 was also used for figure design. A confidence level of 95% was set as the basis for determining the significance difference.

## Results and discussion

### Effect of the experimental model on the different responses

The Box–Behken design applied is presented in Table 2 with the different conditions of temperature, extraction time and solute/solvent. The different experimental conditions evaluated had a significant effect (*p* < 0.05) on the evaluated responses: total polyphenols (TPC), total flavonoids (TFC) and antioxidant activities (DPPH and FRAP). Indeed, TPC levels varied from 5.25 to 10.58 gGAE/100gDW while flavonoids varied from 0.28 to 0.81 gQE/100gDW. A concentration ranging from 0.24 to 0.70 mg/mL could scavenge 50% of DPPH free radicals while the antioxidant power of ferric reduction ranged from 2.4 to 6.55 gAAE/100gDW. These optimal results are similar to those obtained by Ochoa-velascho et al.^23^ on the extraction kinetics of total phenolic compounds (TPC), total flavonoids (TFC) and total antioxidant capacity (TAC) under the following experimental conditions: Temperatures (50, 60, 70 or 80 °C) and at different solvent-product weight ratios (100:1, 200:1 or 300:1 g/g). This difference could be explained by the extension of the experimental model to a third experimental factor: the extraction time. Furthermore, Haspari et al.^24^ revealed that the solute–solvent ratio, the extraction temperature and the extraction time are the major parameters influencing the extraction efficiency of phenolic compounds, flavonoids and their antioxidant activities. According to Noroozi et al.^25^ continuous extraction assisted by ultrasound increases the levels of antioxidant compounds by weakening the cell membranes and facilitates the extraction of these bioactive compounds. Furthermore, other studies have shown that the main phenolic compounds involved are gallic acid, chlorogenic acid, p-coumaric acid, ferulic acid and apigenin among others^16, 26^. According to some authors, in addition to phenolic compounds, continuous ultrasound extraction has revealed to be a good heteropolysaccharide extractor^27^ while Bimakr et al.^28^ suggests that the sonicator has a limitation in extracting lipids compared to the shoxlet. Thus, continuous extraction with the sonicator should be more recommended for non-lipid compounds, mainly antioxidant compounds. The analysis of variance of the different independent factors on the responses were also recorded (Table 4). Indeed, the sums of squares, the p-values (A: solvent ratio, B: temperature and C: extraction time), the quadratic expressions (A^2^, B^2^, C^2^) as well as the interaction of variables (AB, AC, BC) are the main variables studied.

**Table 2:** Experimental conditions for Box–Behnken design and the corresponding results obtained for TPC, TFC, FRAP and DPPH.

Run	Ratio (mg/mL)	Temperature (°C)	Duration (min)	TPC (gGAE/100gDW)	TFC (gQE/100gDW)	IC_50_-DPPH (mg/mL)	FRAP (gAAE/100gDW)
1	33	40	60	7.65	0.48	0.7	3.6
2	50	40	60	8.34	0.38	0.69	3.82
3	33	60	60	7.9	0.42	0.52	4.7
4	50	60	60	8.03	0.39	0.48	6.31
5	33	50	30	6.78	0.47	0.51	3.8
6	50	50	30	8.06	0.38	0.32	5.51
7	33	50	90	6.37	0.71	0.5	4.7
8	50	50	90	7.44	0.44	0.63	2.4
9	41.50	40	30	5.25	0.28	0.5	3.71
10	41.50	60	30	10.09	0.6	0.43	4.25
11	41.50	40	90	10.55	0.51	0.43	4.31
12	41.50	60	90	8.79	0.52	0.52	4.24
13	41.50	50	60	10.52	0.75	0.26	5.57
14	41.50	50	60	10.2	0.81	0.29	5.85
15	41.50	50	60	10.58	0.79	0.24	5.77
16	41.50	50	60	9.42	0.76	0.32	6.1
17	41.50	50	60	9.48	0.65	0.34	6.55

### Influence of operational parameters on the content of bioactive compound: TPC and TFC

**Figure 1:**
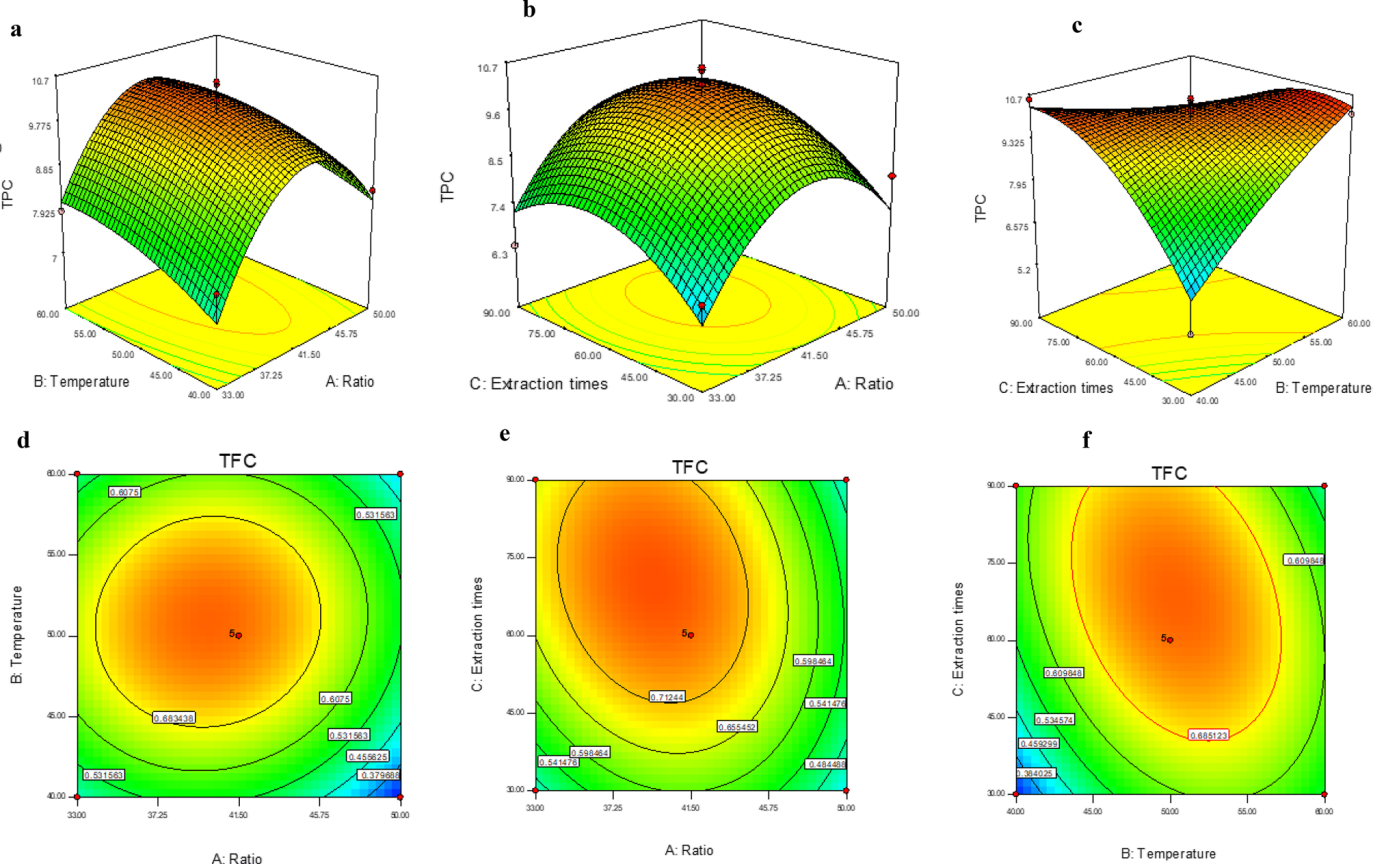
Response and contour plots showing the effect of ratio (**A**), extraction temperature (**B**), extraction time (**C**) on total polyphenols and flavonoids content.

Table 4 showed that the quadratic model used, all quadratic coefficients (A^2^, B^2^, and C^2^), and the interaction coefficient BC were significant in the model developed for total phenolic content (*p* < 0.05). However, the linear effects (A, B and C) were not significant (*p* > 0.05). To express the significance of the quadratic model used, a regression equation was developed (Table 3). In addition, the high R^2^ (0.85) and Adj-R^2^ (0.66), the coefficient of variation CV (10.76) and the non-significant value for lack of fit (4.69) confirmed that the mathematical model of equation (Y1) was adequate to predict the total phenolic content according to the various combination of variables values. These results corroborate those of Iftikhar et al.^29^ on rye bran matrices. Indeed, Fig. 1a shows that gentle heating contributes to the softening of plant tissues, especially the cell wall, and to the hydrolysis of the bonds between phenolic compounds and cellular components (phenol–protein or phenol-poly-saccharide). As a result, the extracted phenolic yield would be higher^30^. The diffusion process takes place due to the concentration gradient, according to Fick's law. Thus, a high concentration contributes to a saturation of the reaction medium and a low diffusion of the extraction solvent^31^. Figure 1b, c show that a long extraction time contributes to an improved phenolic profile. Due to the extensive contact between the solvent and the solid material, the extractability of phenolic compounds is improved by diffusion or destruction of the cell wall^32^. However, high temperature for a long time could reduce the phenolic content due to polymerisation reactions between the compounds^33^.

**Table 3:** Designed equation models for the selected dependent variables Responses.

Responses	Equations	R^2^	R^2^-adj	% C. V
TPC	$$\begin{aligned} {\text{Y1}} = & - 68.75 \, + 2.19 \,{\mathbf{R}} + 0.71 \,{\mathbf{T}} + 0.44 \,{\mathbf{E}}{-} 0.02 \,{\mathbf{R}}^{{2}{}} {-} 0.0027\,{\mathbf{T}}^{2} \\ & {-} 0.0012 \,{\mathbf{E}}^{2} {-} 0.0016\,{\mathbf{R}}*\,{\mathbf{T}}{-} 0.0002 \,{\mathbf{R}}*\,{\mathbf{E}}{-} 0.0055 \,T*\,{\mathbf{E}} \\ \end{aligned}$$	0.85	0.66	10.76
TFC	$$\begin{aligned} {\text{Y2}} = & - {8}.{59 } + 0.{172} \,{\mathbf{R}} + 0.{189} \,{\mathbf{T}} + 0.0{35} \,{\mathbf{E}}{-} 0.00{21} \,{\mathbf{R}}^{\,{\mathbf{2}}} - 0.00{178} \,{\mathbf{T}}^{\,{\mathbf{2}}} \\ & - 0.000{1} \,{\mathbf{E}}^{\,{\mathbf{2}}} + 0.000{2} \,{\mathbf{R}}*\,{\mathbf{T}}{-} 0.000{1}\,{\mathbf{R}}*\,{\mathbf{E}}{-} 0.000{25} \,{\mathbf{T}}*\,{\mathbf{E}} \\ \end{aligned}$$	0.90	0.78	14.22
DPPH	$$\begin{aligned} {\text{Y3}} = & {9}.{14 }{-} 0.{2}0{41} \,{\mathbf{R}}{-} 0.{1527} \,{\mathbf{T}}{-} 0.0{2318} \,{\mathbf{E}} + 0.00{22}\,{\mathbf{R}}^{\,{\mathbf{2}}} + 0.00{14}\,{\mathbf{T}}^{\,{\mathbf{2}}} \\ & + 0.0000{4}\,{\mathbf{E}}^{\,{\mathbf{2}}} {-} 0.0000{88}\,{\mathbf{R}}*\,{\mathbf{T}} + 0.000{3} \,{\mathbf{R}}*\,{\mathbf{E}} + 0.000{1}\,{\mathbf{T}}*\,{\mathbf{E}} \\ \end{aligned}$$	0.88	0.73	16.23
FRAP	$$\begin{aligned} {\text{Y4}} = & - {38}.{15} + 0.{857} \,{\mathbf{R}} + 0.{5893} \,{\mathbf{T}} + 0.{3385}\,{\mathbf{E}}{-} 0.00{97} \,{\mathbf{R}}^{\,{\mathbf{2}}} {-} 0.00{67} \,{\mathbf{T}}^{\,{\mathbf{2}}} \\ & {-} 0.00{13} \,{\mathbf{E}}^{\,{\mathbf{2}}} + 0.00{4} \,{\mathbf{R}}*\,{\mathbf{T}} - 0.00{39} \,{\mathbf{R}}*\,{\mathbf{E}} - \, 0.000{5} \,{\mathbf{T}}*\,{\mathbf{E}} \\ \end{aligned}$$	0.83	0.61	15.04

*R* ratio, *T* temperature, *E* extraction time, *%CV* coefficient of variation.

Analysis of variance (ANOVA, *p* < 0.05) for total flavonoid content showed that the model and quadratic coefficients (A, B, and C) were significant, in contrast to their interaction (Table 4). In addition, they had a significant correlation coefficient (R^2^ = 0.90) and adequate experimental data for the estimated model (no fit, *p* > 0.05) (Tables 3,4), suggesting an approximation of a real system^34^. Total flavonoid content is optimized by elevated temperature, long extraction time and unsaturated solvent–solute concentration (Fig. 1d–f**)**. However, the extraction of flavonoids is optimal for a combination of ultrasonic effects with cavitation, low to medium temperatures (between 20 and 70 °C), due to the electro sensitivity of these bioactive molecules^35^.

**Table 4:** Analysis of variance for the independent variables studied in the extraction of Roselle by the experimental treatments.

Source	TPC		TFC		DPPH		FRAP	
	Sum of squares	*p* > F	Sum of squares	*p* > F	Sum of squares	*p* > F	Sum of squares	*p* > F
Model	34.64	0.0293*	0.40	0.0077*	0.29	0.0143*	18.13	0.0434*
A-Ratio	1.26	0.2629	0.030	0.0622	1.513E−003	0.6122	0.19	0.5614
B-Temperature	1.14	0.2842	9.800E−003	0.2458	0.017	0.1176	2.06	0.0861
C-Extraction times	1.10	0.2916	0.025	0.0812	0.013	0.1667	0.33	0.4520
AB	0.078	0.7699	1.225E−003	0.6678	2.250E−004	0.8437	0.48	0.3661
AC	0.011	0.9124	8.100E−003	0.2873	0.026	0.0654	4.02	0.0270*
BC	10.89	0.0089*	0.024	0.0878	6.400E−003	0.3113	0.093	0.6842
A^2^	13.40	0.0054*	0.10	0.0046*	0.11	0.0025*	2.08	0.0850
B^2^	0.32	0.5576	0.13	0.0022*	0.087	0.0050*	1.93	0.0944
C^2^	5.04	0.0449*	0.039	0.0398*	5.533E−003	0.3441	5.89	0.0118*
Residual	5.93		0.043		0.038		3.62	
Lack of fit	4.69	0.0766	0.027	0.2087	0.031	0.0574	2.93	0.0640
Pure error	1.25	-	0.015	-	6.800E−003	-	0.69	-
Cor total	40.57	-	0.45	-	0.32	-	21.75	-

### Influence of operational parameters on antioxidant activities

**Figure 2:**
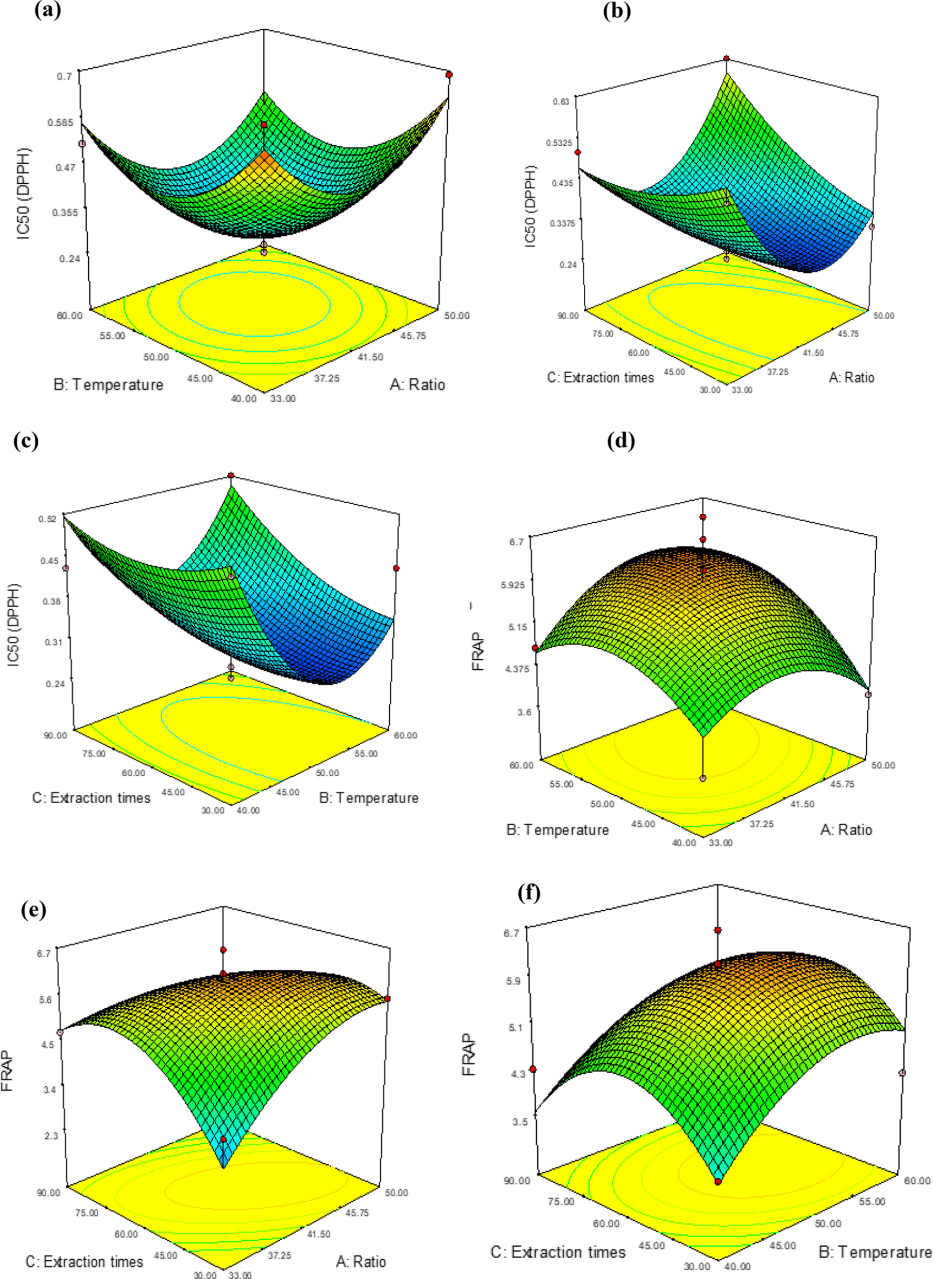
Response plots showing the effect of ratio (**A**), extraction temperature (**B**), extraction times (**C**) on DPPH and FRAP antioxidant activities.

Two estimation methods (DPPH and FRAP) were used for the assessment of antioxidant activity^36, 37^. The quadratic model used was associated with a regression equation recorded in Table 3. Furthermore, the high values of R^2^ (0.88) and Adj-R^2^ (0.73), the low value of CV (16.23) and the insignificant value of lack of fit (0.031) confirmed that the mathematical model of the equation (Y3) was adequate to predict the content of DPPH antioxidant activity according to the different values of combination of the variables (Table 2). The variables studied: solute/solvent ratio, sonication temperature and extraction time, influenced the DPPH antioxidant activity of roselle extracts. For an extraction time set at 50 °C, the effect of concentration and temperature positively influenced the inhibition of DPPH by roselle extract (Fig. 2a). However, at a temperature of 57 °C and a concentration of 47 mg/mL, we note a degradation of the antioxidant activity of roselle extracts (Fig. 2a). A low solute to solvent ratio under low extraction temperature presented less interesting DPPH antioxidant activities. For the optimum ratio and temperature levels, an extraction time of around 1 h increased the DPPH radical inhibitory activity (Fig. 2b, c). On the other hand, DPPH radical scavenging activities decrease with longer ultrasonic irradiation time. These studies clearly show that the ratio, temperature modifies the DPPH activity positively in the middle region and then follows a negative trend for longer extraction times. The quadratic model applied for iron reducing power had a significant effect (*p* < 0.05) on seed FRAP. For this purpose, quadratic coefficients and the significant interactions are consigned (Table 4). A regression model (Y4) was developed with R^2^ = 0.83, Adj-R^2^ = 0.61 and CV = 15.04. The 3D Box–Behkhen response surface plots showed the FRAP antioxidant activity varies positively for the average temperature and extraction period (Fig. 2d–f). In other words, the reducing antioxidant power of iron is interesting under mild heating and at medium time and decreases under high temperature for long extraction time were above average. This could be due to two reasons: (1) thermal degradation of antioxidant substances, including TPC, TFC and other thermolabile compounds^38^; (2) a direct reduction in antioxidant power due to heat and the presence of oxygen in the reaction matrix^39^.

### Optimal condition of extractions variables

**Figure 3:**
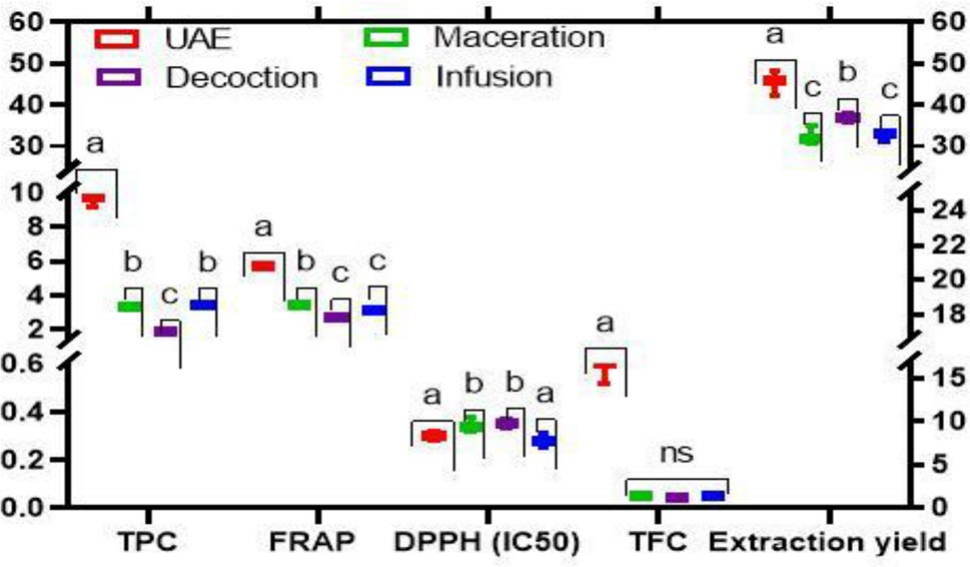
Comparative study between conventional extraction methods and UAE. The different levels of TPC; TFC, FRAP and IC50 DPPH are associated with the Y-axis on the left while the extraction yield is associated with the X-axis on the right. *ns* non-significant difference; and all factors sharing no letters in common are significantly different at *p* value (*p* ≤ 0.05).

In order to achieve the maximum levels of extraction efficiency of phenolic compounds and their antioxidant activities, an experimental method of ultrasound assisted extraction was carried out. Different temperatures (40–60 °C), for an extraction time fluctuating from 30 to 90 min and roselle matrices whose concentration (m/v) was set at values ranging from 33 to 50 mg/mL. The optimum conditions determined from a Box–Bohkehn surface response indicate a ratio of 41.81 mg/mL at a temperature of 52.35 °C for 57.77 min (Table 5). Therefore, the predicted and experimental values at this extraction condition are recorded in Table 5 with a desirability of 0.89. Thus, for an industrial application, this optimization model is technologically, financially and energetically viable as it requires a reasonable concentration, time and temperature. A comparison of this ultrasonic optimal condition with conventional methods showed a better extraction yield, a higher content of total polyphenol and total flavonoids and a FRAP antioxidant power (Fig. 3). Indeed, conventional methods such as decoction, infusion and maceration have several extractive limitations including poor solvent diffusion in the matrix and degradation of the solvent^40, 41^.

**Table 5:** Experimental values and predicted values of response variables at optimum extraction conditions.

Response variables	Optimum extractions conditions			Maximum value	
	A	B	C	Predicted values	Experimental values
TPC	41.81 mg/mL	52.35 °C	57.77 min	10.11	9.81
TFC	41.81 mg/mL	52.35 °C	57.77 min	0.74	0.68
FRAP	41.81 mg/mL	52.35 °C	57.77 min	6.09	5.67
DPPH	41.81 mg/mL	52.35 °C	57.77 min	0.28	0.30

*A* ratio, *B* temperature, *C* time of extraction. Desirability: 0.89.

## Conclusion

The development of appropriate extraction technology for various plant matrices is a requirement for food and pharmaceutical industries. In this study, a Box–Behnken design of a response surface allowed to establish an optimal extraction condition integrating three independent variables: temperature, solute–solvent ratio and extraction time. Thus, the experimental data obtained are interesting for the different response factors. Furthermore, these results show that ultrasound assisted extraction is an alternative to conventional methods. An extension to other types of responses including anthocyanins and colorimetry would be an asset for industrial application at the scale of natural colorant production.

## Data Availability

The data used to support the findings of this study are available from the corresponding author upon request.
